# Supplementary material for: Platelet-derived exerkine CXCL4/platelet factor 4 rejuvenates hippocampal neurogenesis and restores cognitive function in aged mice
Source: Nat Commun. 2023 Aug 16;14:4375. doi: 10.1038/s41467-023-39873-9 (PMC10432533; doi:10.1038/s41467-023-39873-9)
Supplement: Supplementary file 1 — Supplementary Information [file 41467_2023_39873_MOESM1_ESM.pdf]

Supplementary information

A platelet-derived exerkine rejuvenates hippocampal neurogenesis and restores cognitive function in the aged brain

Odette Leiter, David Brici, Stephen J. Fletcher, Xuan Ling Hilary Yong, Jocelyn Widagdo, Nicholas Matigian, Adam B. Schroer, Gregor Bieri, Daniel G. Blackmore, Perry F. Bartlett, Victor Anggono, Saul A. Villeda, and Tara L. Walker

Correspondence to: t.walker1@uq.edu.au

This PDF file includes: Supplementary figures 1 – 12
Supplementary table 1

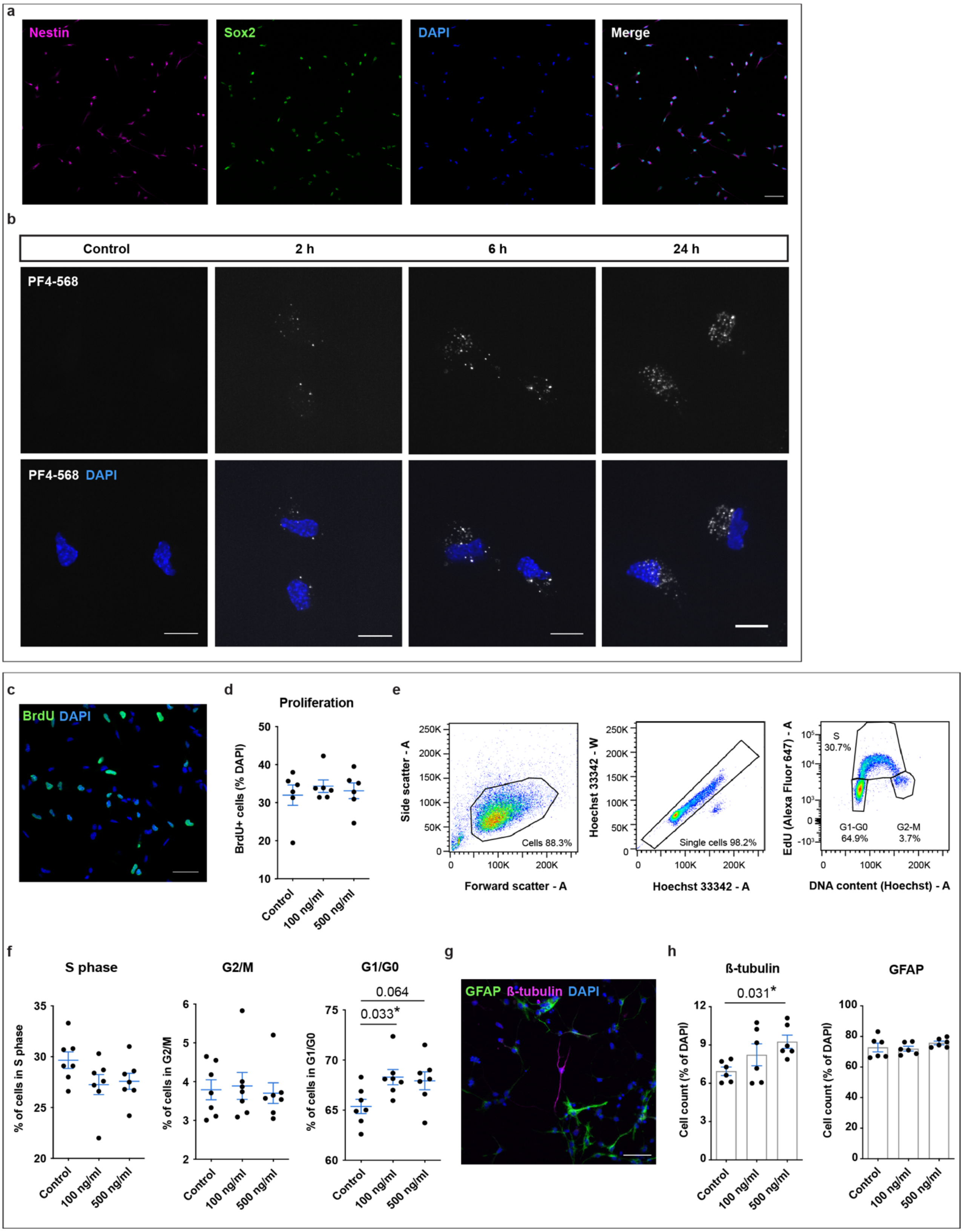

Supplementary fig. 1 | PF4 promotes neuronal differentiation, but not proliferation of adult neural precursor cells *in vitro*. a, Representative image of adult neural precursor cells growing as an adherent monolayer culture 48h post seeing, stained for the neural precursor cell markers nestin (magenta) and Sox2 (green). Scale bar: 50 µm. **b,** Representative images of neural precursor cells

treated with AlexaFluor-568-labelled recombinant PF4 protein for 2, 6 or 24 h. Representative of six independent experiments. Scale bars: 10 µm. **c,** Representative image of BrdU-labelled neural precursor cells. Scale bar: 50 µm. **d,** PF4 treatment did not change the number of BrdU-labelled cells (n = 6 independent experiments). **e,** Representative flow cytometry plots of the click-iT EdU proliferation assay. A – area, W – width. **f,** PF4 treatment increased the proportion of neural precursor cells present in the G1/G0 phases of the cell cycle (n = 7 independent experiments). **g,** Representative image of differentiated neural precursor cells. Scale bar: 50 µm. **h,** PF4 treatment increased the number of cells that differentiated into β-tubulin$^+$ neurons without affecting GFAP$^+$ astrocyte differentiation (n = 6 independent experiments). Graphs represent mean $\pm$ SEM. Statistical analysis was performed using one-way ANOVAs with Dunnett's post hoc comparison. $^*p < 0.05$. Source data are provided as Source Data file.

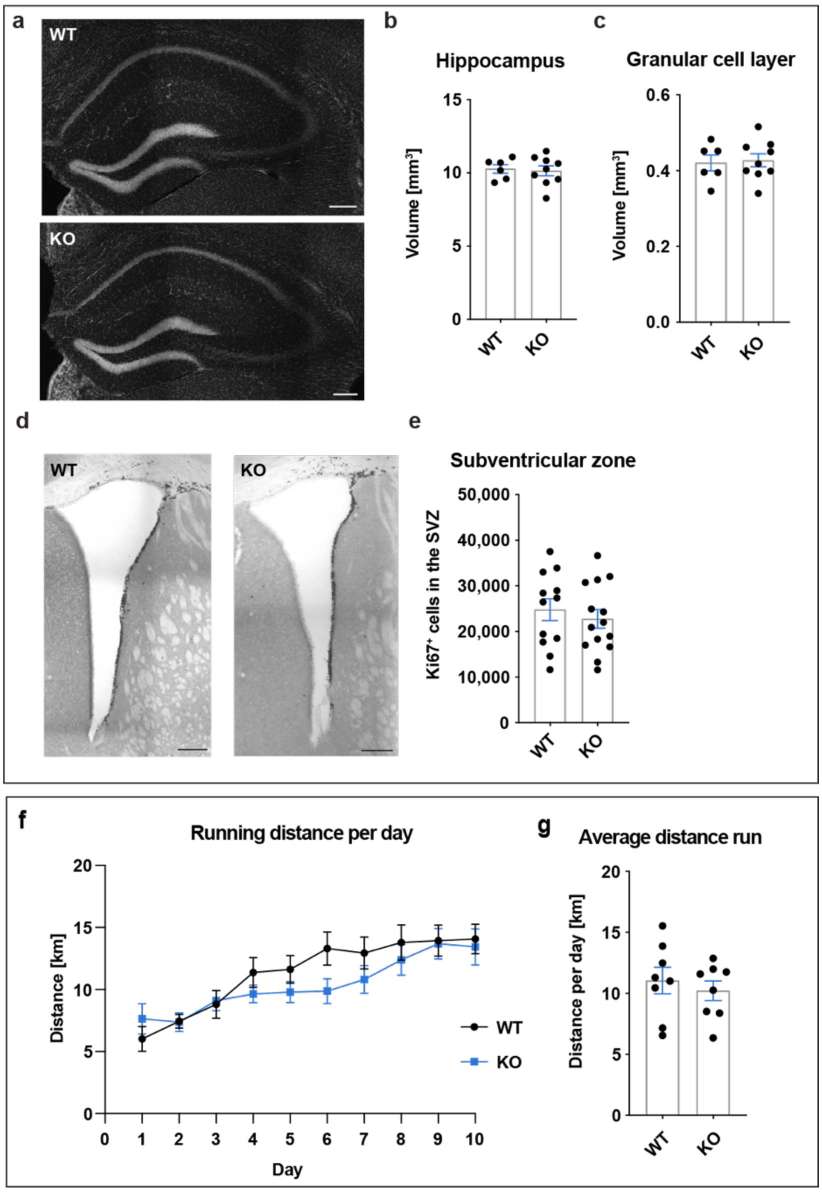

Supplementary fig. 2 | PF4 knockout does not affect hippocampal volume, proliferation in the subventricular zone or running behaviour of 8-week-old mice. a, Representative image of the hippocampus region in 8-week-old PF4 knockout (KO) and wildtype (WT) littermates. DAPI staining, scale bars: 150 µm. **b, c,** Measurements of hippocampal volume **(b)** and volume of the granular cell layer **(c)** revealed no differences between PF4 KO and WT animals. n = 6 WT mice and n = 9 PF4 KO mice. **d,** Representative images of Ki67$^+$ proliferating cells in the subventricular zone (SVZ) of PF4 KO and wildtype littermates. Scale bars: 200 µm. **e,** Quantification of Ki67$^+$ proliferating cells in the SVZ revealed no difference between PF4 KO mice and wildtype littermates. n = 12 WT mice and n = 14 PF4 KO mice. **f,** PF4 KO or WT animals were housed in cages with ad libitum access to a running wheel for a period of 10 days during which the running distance of individual mice was monitored. **g,** Average running distance during the experiment. n = 8 mice per group. Bars represent mean $\pm$ SEM. Source data are provided as Source Data file.

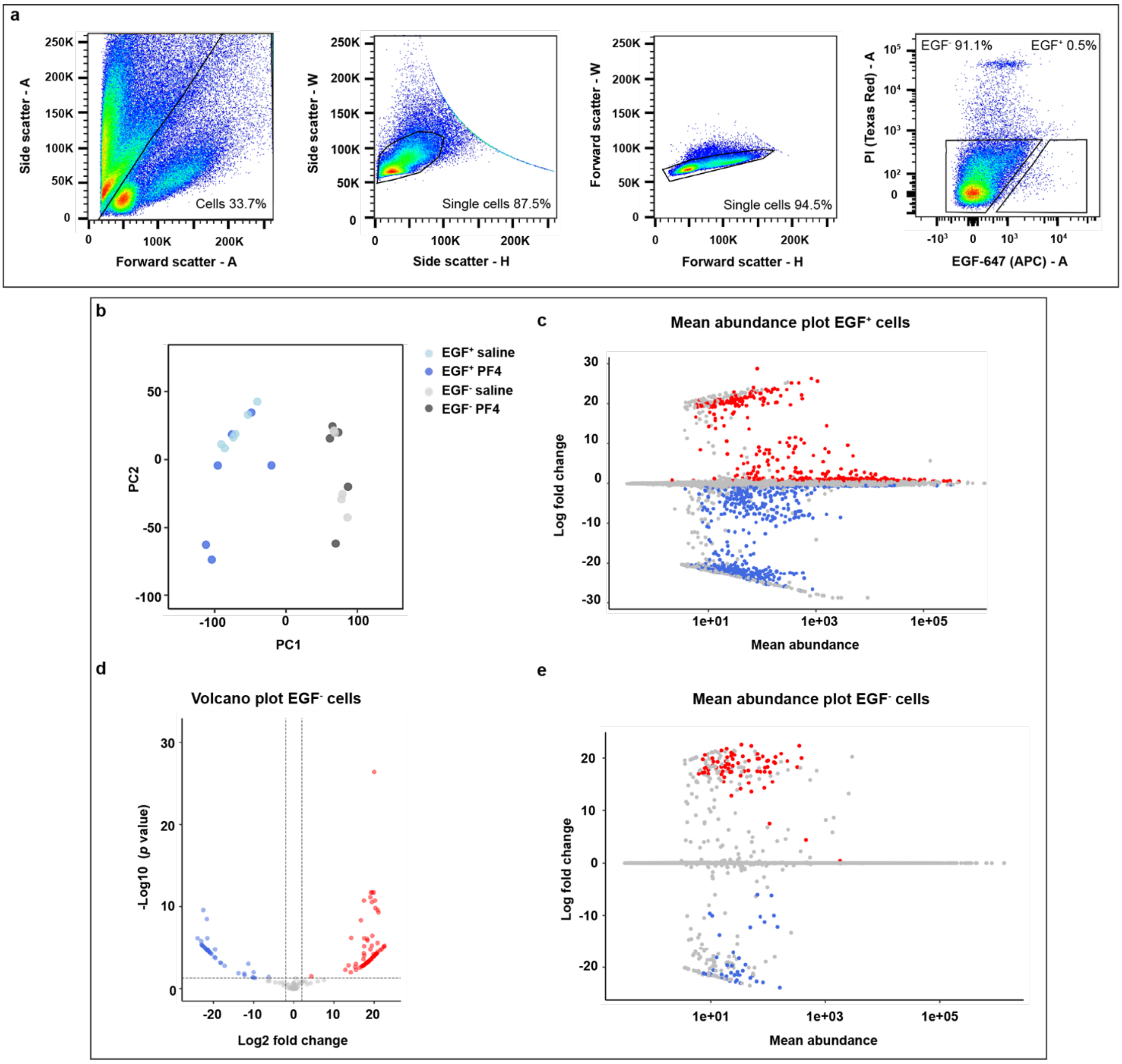

Supplementary fig. 3 | RNA sequencing of adult neural precursor cells following PF4 treatment. a, Representative flow cytometry plots. Cells were first separated from debris using side scatter area and forward scatter area (left panel), followed by the exclusion of doublets using side scatter width and side scatter height and forward scatter width and forward scatter height (middle panels). Cells positive or negative for epidermal growth factor (EGF) conjugated to AlexaFluor 647 were then collected for immediate RNA isolation (right panel). **b,** A principal-component (PC) analysis showed distinct clusters of EGF^+ and EGF^- cells. **c,** Mean abundance plot showing differentially expressed genes between PF4- and saline-treated EGF^+ cells, with upregulated genes in red (log_2 fold change > 2 and adjusted p value < 0.05) and downregulated genes in blue (log_2 fold change < -2 and adjusted p value < 0.05). Wald test and Benjamini-Hochberg correction. **d,** Volcano plot of differentially expressed genes in EGF^- dentate gyrus cells treated with PF4 and saline, with significantly upregulated genes in red (log_2 fold change > 2 and adjusted p value < 0.05) and significantly downregulated genes in blue (log_2 fold change < -2 and adjusted p value < 0.05). Wald test and Benjamini-Hochberg correction. **e,** Mean abundance plot showing differentially expressed genes between PF4- and saline-

treated EGF$^-$ dentate gyrus cells, with upregulated genes in red (log_2 fold change > 2 and adjusted *p* value < 0.05) and downregulated genes in blue (log_2 fold change < -2 and adjusted *p* value < 0.05). Wald test and Benjamini-Hochberg correction. Source data are provided as Source Data file.

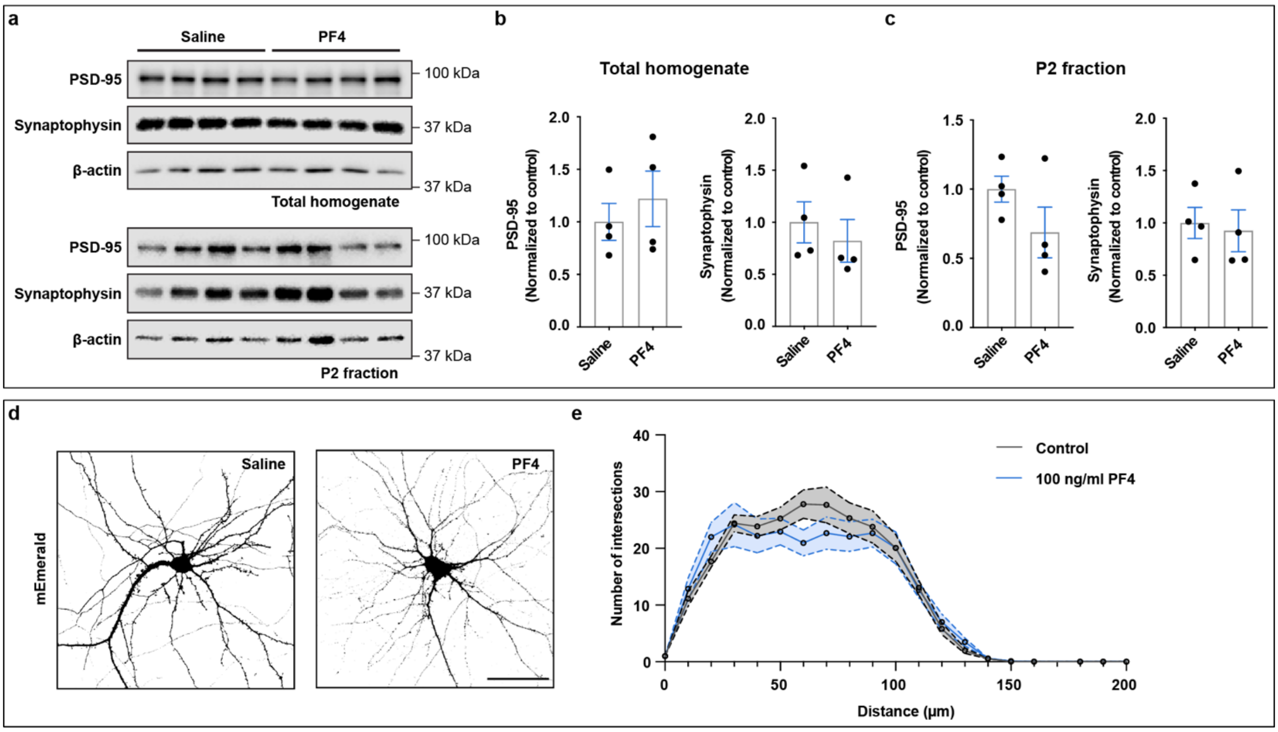

Supplementary figure 4 | PF4 treatment does not affect the expression of PSD-95 or synaptophysin in the dentate gyrus or the dendrite complexity of mature neurons. a, 8-week-old C57BL/6J mice were injected with saline (vehicle control) or 5 μg/ml PF4, every second day for 1 week. Dentate gyrus tissues were collected and subjected to subcellular fractionation. The total homogenate and the crude synaptosomes (P2 fraction) were subjected to western blotting and probed with specific antibodies against PSD-95 (postsynaptic marker), synaptophysin (presynaptic marker) and β-actin. **b, c,** The levels of PSD-95 and synaptophysin expression in the total homogenate **(b)** and P2 fraction **(c)** were quantified by normalizing them with β-actin. Data represent mean ± SEM (n = 4 mice per group). **d,** Cultured hippocampal neurons were transfected with a plasmid encoding the cell-fill marker mEmerald at days *in vitro* (DIV) 13. At DIV 16, neurons were treated with either saline or 100 ng/ml PF4 for 24 h. Neurons were fixed and imaged on a confocal microscope to visualize the morphology of the neurons through the mEmerald fluorescence. Scale bar, 50 μm. **e,** Neurite complexity was quantified using Sholl analysis. Concentric shells with increasing radii were superimposed on the mEmerald fluorescence images of neurons, and the number of processes intersecting each sampling shell was counted after thresholding. Data represent mean ± SEM (n = 15 neurons per group). Uncropped gels are depicted in this document in Supplementary fig. 12. Source data are provided as Source Data file.

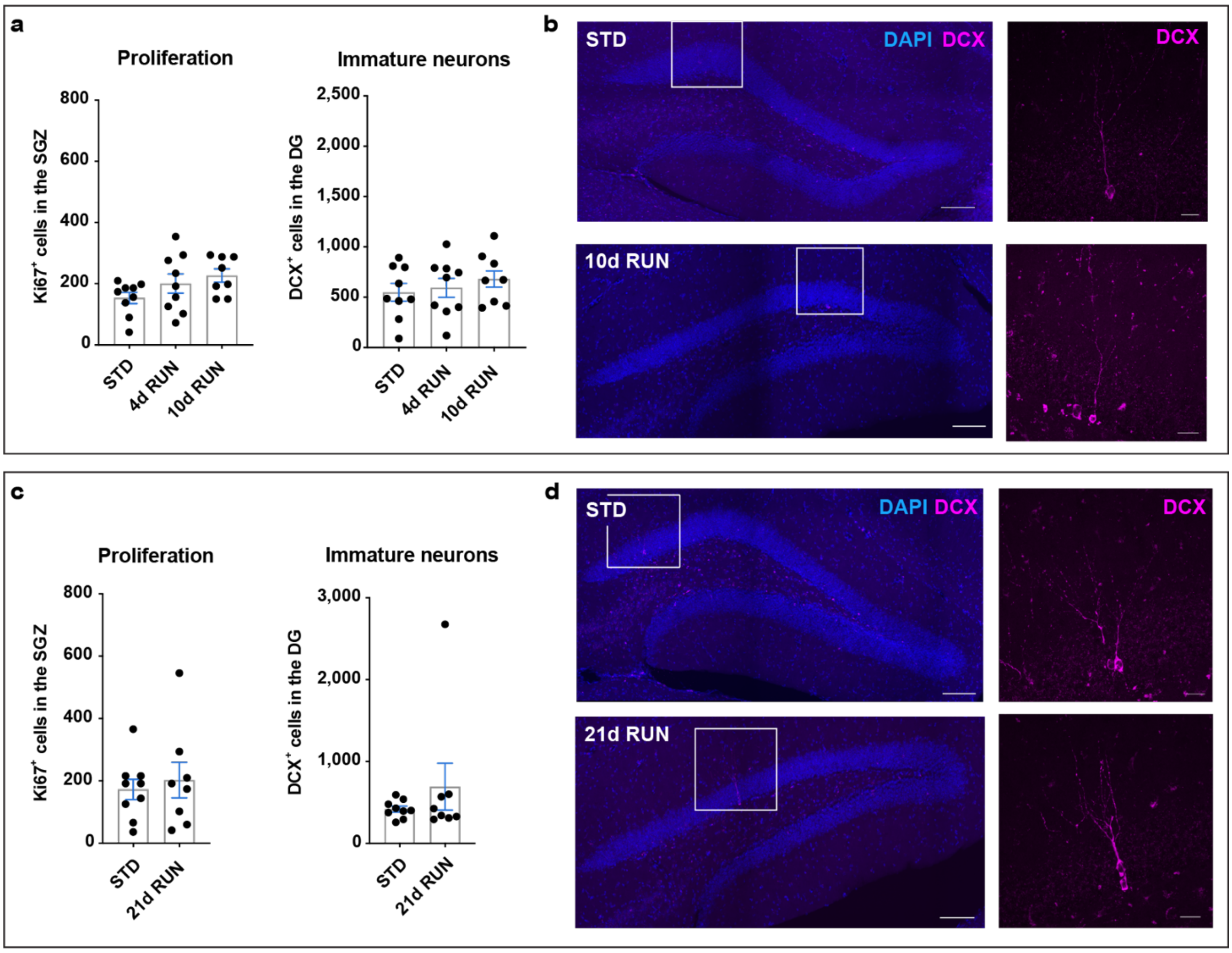

Supplementary fig. 5 | Short and intermediate exercise paradigms do not increase hippocampal neurogenesis in 18-month-old C57BL/6J mice. a, The number of proliferating Ki67$^+$ cells and DCX$^+$ immature neurons does not differ from standard-housed age-matched controls following 4 (4d RUN) or 10 (10d RUN) days of exercise. **b,** Representative images of DCX$^+$ cells in the dentate gyrus of 18-month-old mice following standard-housing or 10 days of exercise. Scale bars: 100 µm (left) and 20 µm (right). **c,** 21 days of exercise (21d RUN) does not affect the number of proliferating Ki67$^+$ cells or DCX$^+$ immature neurons. **d,** Representative images of DCX$^+$ cells in the dentate gyrus of 18-month-old mice following standard-housing or 21 days of exercise. Scale bars: 100 µm (left) and 20 µm (right). SGZ – subgranular zone, DG – dentate gyrus. n = 9 in the standard-housed and 4d RUN groups and n = 8 mice in the 10d and 21d RUN groups. Bars graphs represent mean $\pm$ SEM. Source data are provided as Source Data file.

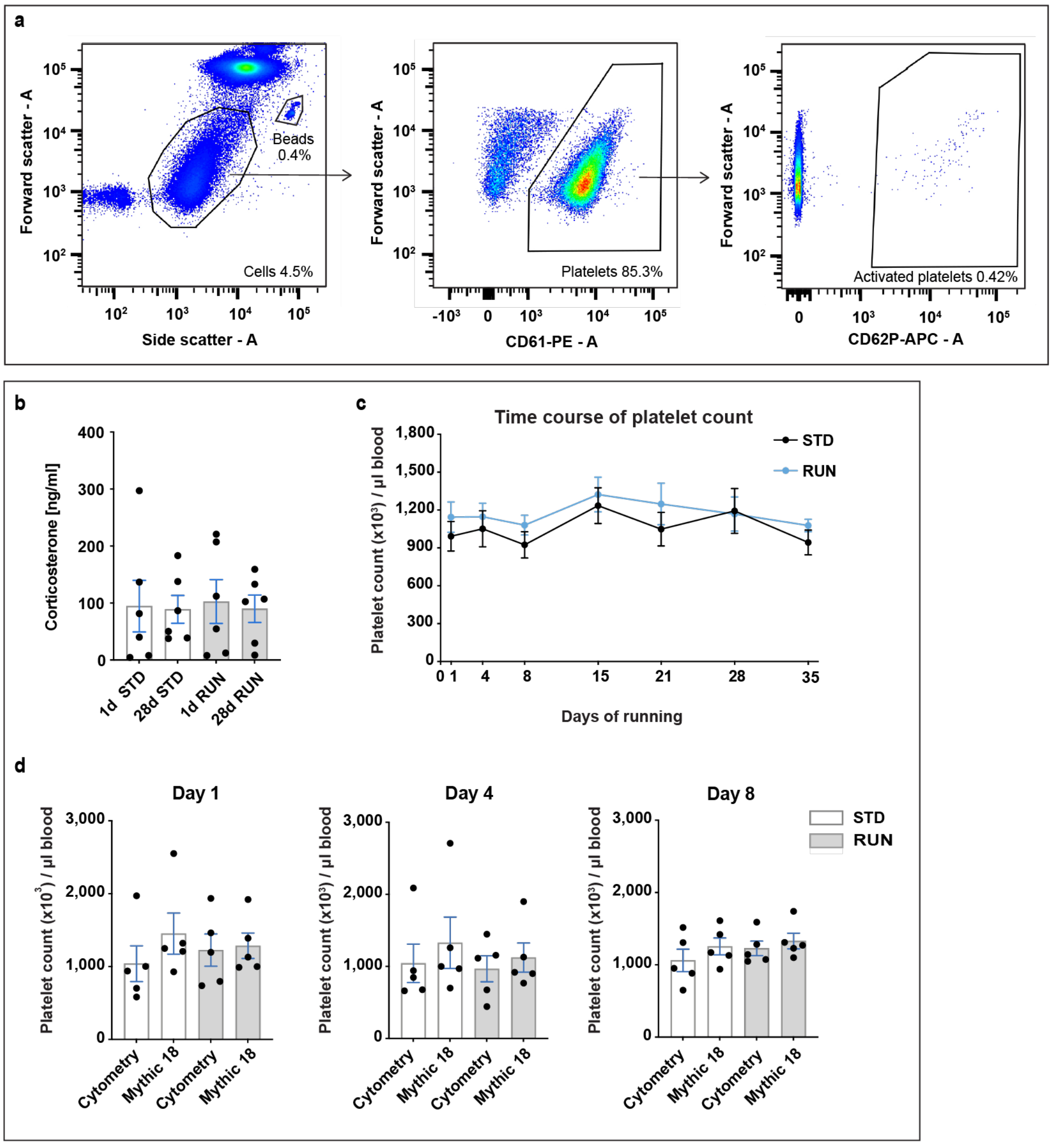

Supplementary figure 6 | Platelet count does not change following exercise. a, Representative flow cytometry plots of platelet count and activation. Left: the platelet population and reference beads were first gated based on forward and side scatter. Platelets and activated platelets were defined by the expression of CD61 (middle) and CD62P (right), respectively. A – area. **b,** Corticosterone levels in the blood of 18-month-old C57BL/6J mice measured by ELISA show no differences at the beginning (d1) or at a prolonged running time point (d28; n = 6 mice per group). **c,** The platelet count of 18-month-old C57BL/6J mice was measured longitudinally over a period of 35 days using flow cytometry, revealing no difference between mice that were housed with or without a running wheel (n = 10 mice per group). **d,** Simultaneous platelet count measurements of a subset of mice on day 1, day 4 and day 8 of the experiment using a Mythic18 Vet automated haematology analyser and flow cytometry revealed no difference between methods (n = 5 mice per group). Bars represent mean ± SEM, with white bars representing standard housing and grey bars depicting running conditions. Source data are provided as Source Data file.

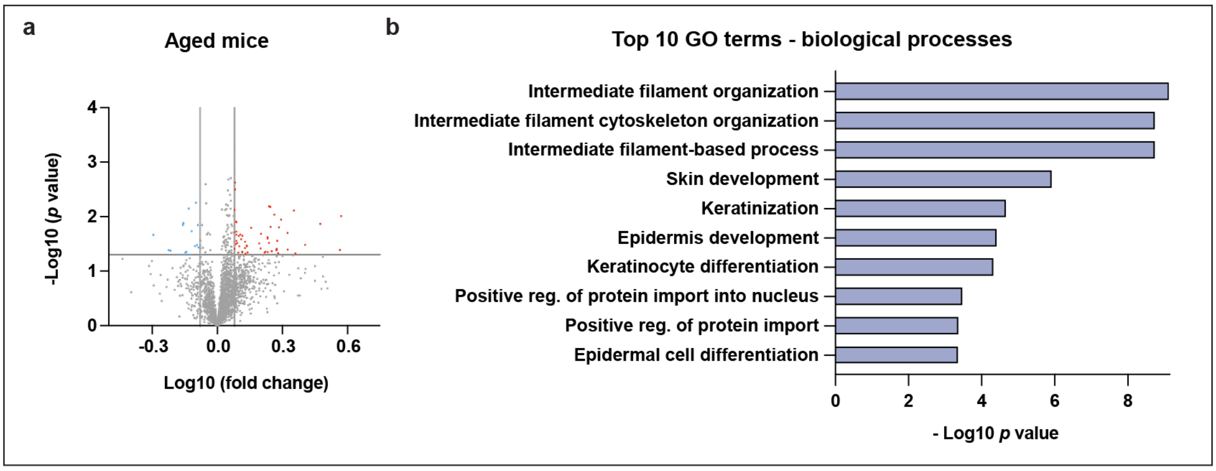

Supplementary figure 7 | Platelet proteomic analysis of aged mice following 28 days of exercise. **a,** Volcano plot of quantified and differentially expressed proteins in the platelet lysate of 20-month-old C56BL/6J mice following 28 days of running. Significantly upregulated proteins are labelled red ($p < 0.05$ and fold change > 1.2) and significantly downregulated proteins are shown in blue ($p < 0.05$ and fold change < -1.2). Pairwise relative-abundance comparison using two-tailed *t*-tests. **b,** Gene ontology (GO) enrichment analysis of significantly increased proteins in the platelet lysate of aged running mice following 28 days of exercise compared to standard-housed mice. Bar graphs show the top 10 significantly enriched GO terms for biological processes ranked by Benjamini-Hochberg false discovery rate-corrected *p* values. Source data are provided as Source Data file.

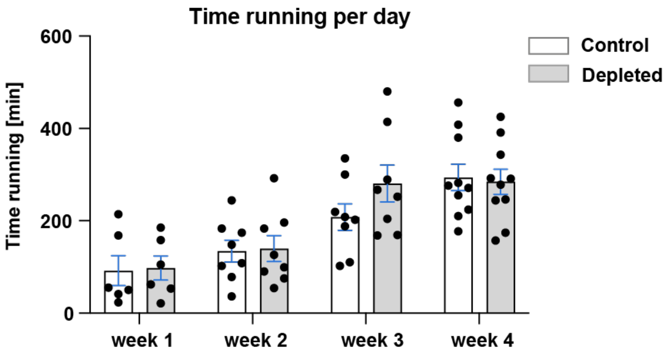

Supplementary fig. 8 | Running behaviour in 18-month-old C57BL/6J mice. 18-month-old C57BL/6J mice received intraperitoneal injections of either antiplatelet serum (depleted) or normal rabbit serum (control) every second day for 30 days, during which a subset of mice was housed in cages with a running wheel for 28 days (housed three mice per cage). Monitoring of their running behaviour revealed no difference between exercising platelet-depleted mice and control animals. Week 1: 0-7 days, tracking data from n = 6 cages; week 2: 8-14 days, tracking data from n = 8 cages; week 3: 15-21 days, tracking data from n = 8 cages; week 4: 22-28 days of exercise, tracking data from n = 10 cages. Bars represent mean ± SEM. Source data are provided as Source Data file.

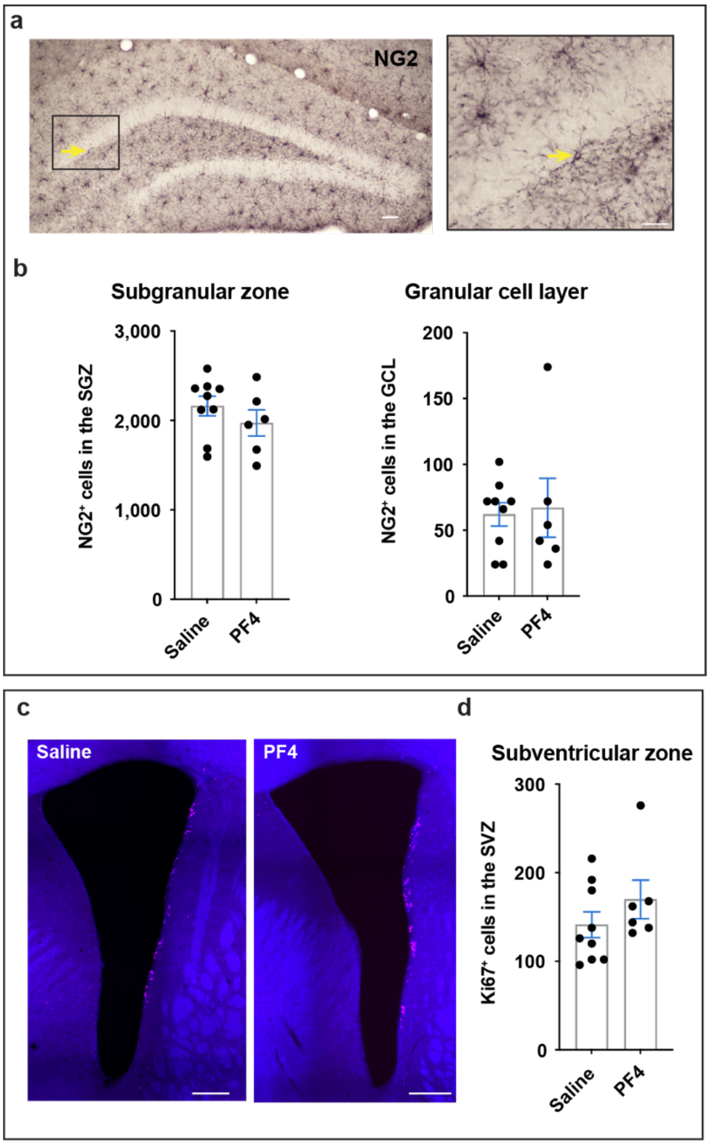

Supplementary fig. 9 | Systemic PF4 treatment of 20-month-old C57BL/6J mice does not change the number of NG2$^+$ cells in the dentate gyrus or proliferating cells in the subventricular zone. 20-month-old C57BL/6J mice received systemic injections of either saline or PF4 (500 ng) every third day for 24 days. **a,** Representative image of NG2$^+$ cells in the hippocampal dentate gyrus (left; scale bar: 50 µm) and subgranular zone (right; scale bar: 25 µm). Yellow arrows mark the same cell at different magnifications. **b,** Quantification of NG2$^+$ cells in the subgranular zone (SGZ) and granular cell layer (GCL) of the dentate gyrus revealed no differences between saline-treated and PF4-treated mice. n = 9 saline-treated mice and n = 6 PF4-treated animals. **c,** Representative images of proliferating Ki67$^+$ cells (magenta) in the subventricular zone (SVZ) of 20-month-old C57BL/6J mice that either received saline or PF4. Scale bars: 200µm. **d,** Quantification of Ki67$^+$ cells revealed no difference in proliferating cells in the SVZ between groups. n = 9 saline-treated mice and n = 7 PF4-treated animals. Bars represent mean $\pm$ SEM. Source data are provided as Source Data file.

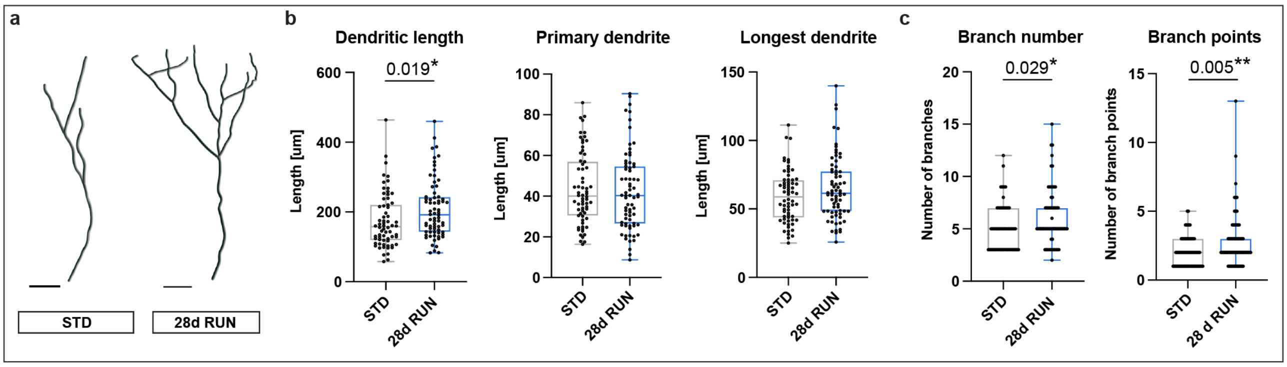

Supplementary fig. 10 | Exercise increases the dendritic complexity of DCX⁺ immature neurons in 18-month-old C57BL/6J mice. a, Representative reconstruction of postmitotic DCX^+ cells in standard-housed (STD) mice and mice housed in cages with a running wheel for 28 days (28d RUN). Scale bars: 15 µm. **b,** Postmitotic DCX^+ cells of mice that ran for 28 days showed an increased total dendritic length compared to those of aged-matched standard-housed control animals. **c,** 28 days of exercise increased the number of dendrites and branch points in postmitotic DCX^+ cells. n = 66 cells from STD mice; n = 71 cells from 28d RUN animals. Box plots show median ± interquartile range, with whiskers defining minimum and maximum. Statistical analysis was performed using unpaired Student's two-tailed *t* tests. *$p < 0.05$, **$p < 0.01$. Source data are provided as Source Data file.

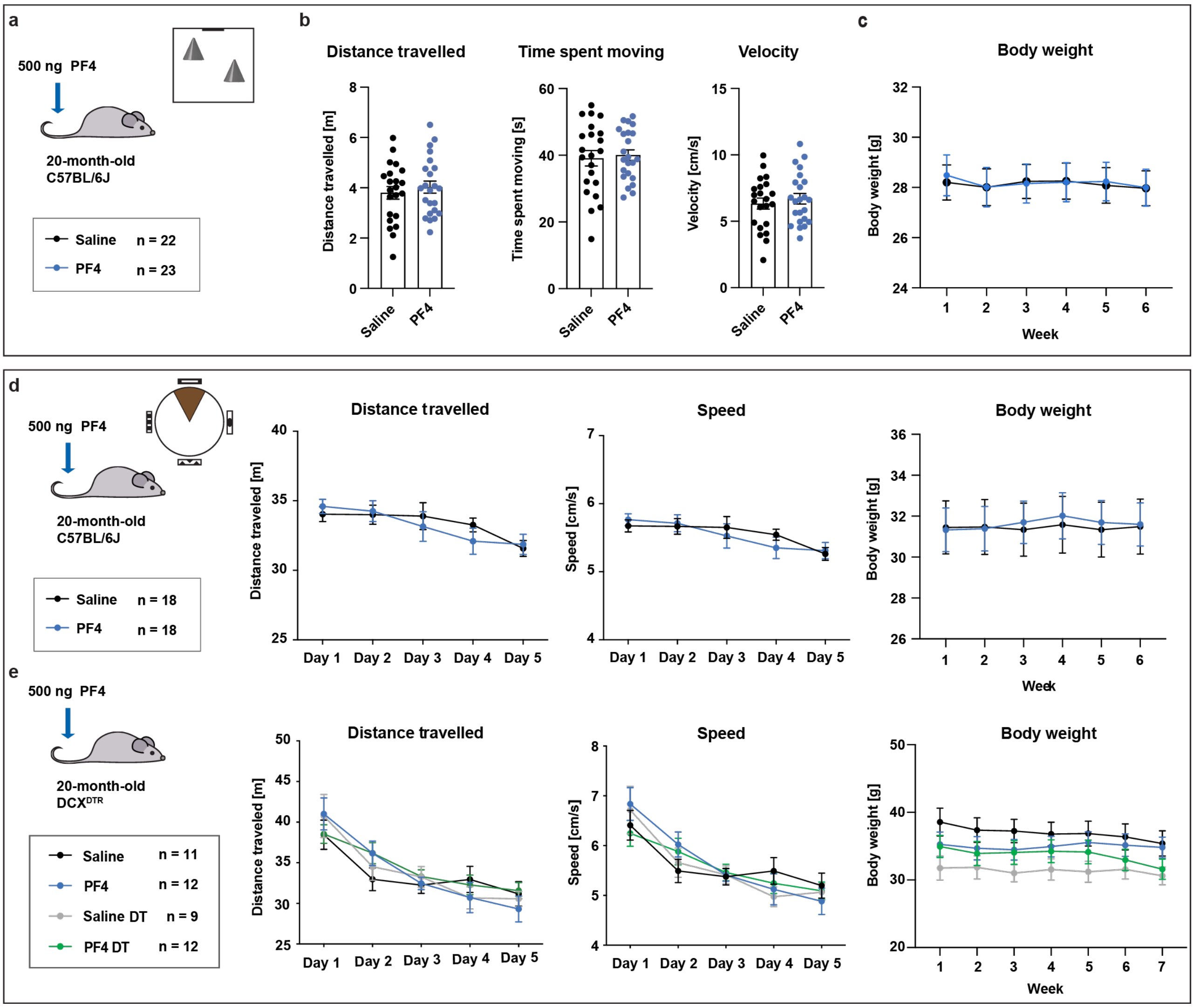

Supplementary fig. 11 | PF4 treatment does not affect movement of the mice during the novel object location and active place avoidance tasks, nor their body weight. a, 20-month-old C57BL/6J mice received systemic PF4 or saline injections every third day for 24 days and were subsequently tested in the novel object location task. **b,** There were no differences in overall movement between groups, including total distance travelled, time spent moving or velocity. **c,** PF4 treatment did not affect the weight of the mice. **d, e,** 20-month-old C57BL/6J mice **(d)** or DCXDTR mice **(e)** received systemic PF4 or saline injections every third day for 24 days and were subsequently tested in the active place avoidance task. The treatment did not cause differences in the total distance travelled or speed, as well as the body weight. Bars represent mean ± SEM. Source data are provided as Source Data file.

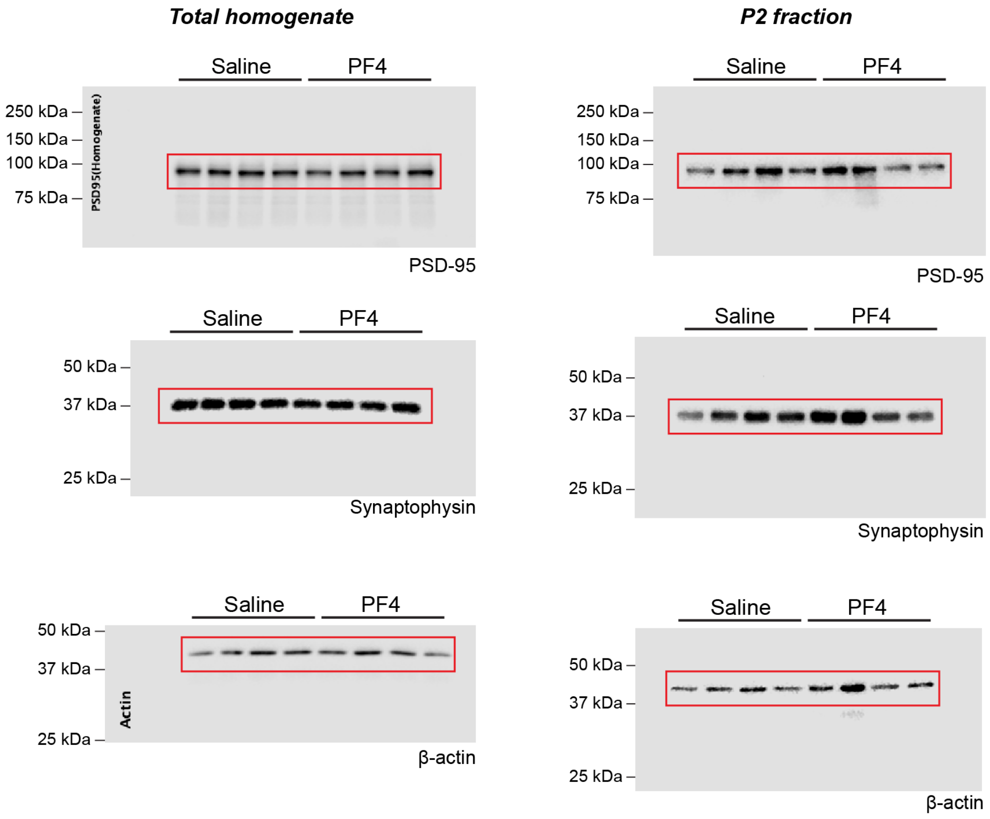

Supplementary fig. 12 | Uncropped gels of western blot analysis. Related to Supplementary figure 4.

Supplementary table 1 | Individual values of platelet count in mice after treatment with anti-mouse platelet serum and control serum.

	STD control serum	RUN control serum	STD anti-platelet serum	RUN anti-platelet serum
Animal 1	1,013	1,269	897	945
Animal 2	868	568	861	1,435
Animal 3	764	765	101	348
Animal 4	1,174	1,182	727	404
Animal 5	1,320	1,058	397	434
Animal 6	1,082	1,473	461	362
Animal 7	1,757	978	289	1,097
Animal 8	1,043	1,730	108	601
Animal 9	1,188	1,256	259	744
Animal 10	1,790	914	36	261
Animal 11	-	763	-	1,384
Animal 12	-	908	-	594
Animal 13	-	1,306	-	437
Animal 14	-	-	-	202
Mean $\pm$ SEM	1,200 $\pm$ 108	1,090 $\pm$ 89	414 $\pm$ 101	661 $\pm$ 108

Data are platelet count ($x10^3$) / ml blood after 30 days of platelet depletion.
