# Supplementary material for: Platelet-derived exerkine CXCL4/platelet factor 4 rejuvenates hippocampal neurogenesis and restores cognitive function in aged mice
Source: Nat Commun. 2023 Aug 16;14:4375. doi: 10.1038/s41467-023-39873-9 (PMC10432533; doi:10.1038/s41467-023-39873-9)
Supplement: Supplementary file 3 — Description of Additional Supplementary Files [file 41467_2023_39873_MOESM3_ESM.pdf]

Description of Additional Supplementary Files

Supplementary Data 1 | Genes determined by ribonucleic acid sequencing analysis of EGF^+ adult neural stem cells following PF4 treatment.

An Excel spreadsheet containing the results of a ribonucleic acid sequencing analysis of EGF^+ cells treated with PF4 compared with EGF^+ cells that were treated with saline. Genes with a fold change greater than ± 1.2 and a *p*-value smaller than 0.05 were considered differentially expressed genes. Upregulated genes following PF4 treatment are highlighted in pink, whereas downregulated genes are marked green. *p*-values lower than 0.05 are highlighted in yellow.

Supplementary Data 2 | Genes determined by ribonucleic acid sequencing analysis of EGF^- dentate gyrus cells following PF4 treatment.

An Excel spreadsheet containing the results of a ribonucleic acid sequencing analysis of EGF^- cells treated with PF4 compared with EGF^- cells that were treated with saline. Genes with a fold change greater than ± 1.2 and a *p*-value smaller than 0.05 were considered differentially expressed genes. Upregulated genes following PF4 treatment are highlighted in pink, whereas downregulated genes are marked green. *p*-values lower than 0.05 are highlighted in yellow.

Supplementary Data 3 | Gene ontology enrichment analysis of differentially expressed genes in EGF^+ adult neural stem cells following PF4 treatment.

An Excel spreadsheet containing results of the gene ontology enrichment analyses performed with gProfiler for upregulated (UP) and downregulated genes (DOWN) in EGF^+ adult neural precursor cells which were treated with PF4 compared to saline-treated controls. BP: biological process; MF: molecular function. CC: cellular compartment, TF: transcription factor.

Supplementary Data 4 | Proteins determined by mass spectrometry analysis of platelet lysate in young running mice.

An Excel spreadsheet containing the results of a mass spectrometry analysis of platelet lysate isolated from young standard-housed and running mice. A pairwise relative-abundance comparison using *t*-tests was performed across both groups. Proteins with a fold change greater than ± 1.2 and a *p*-value smaller than 0.05 were considered differentially expressed proteins. Upregulated proteins following exercise are highlighted in pink, whereas downregulated proteins are marked green. *p*-values lower than 0.05 are highlighted in yellow.

Supplementary Data 5 | Proteins determined by mass spectrometry analysis of platelet lysate in aged running mice.

An Excel spreadsheet containing the results of a mass spectrometry analysis of platelet lysate isolated from aged standard-housed and running mice. A pairwise relative-abundance comparison using *t*-tests was performed across the different groups. Proteins with a fold change greater than ± 1.2 and a *p*-value smaller than 0.05 were considered differentially expressed proteins. Upregulated proteins following exercise are marked pink, whereas downregulated proteins are highlighted in green. *p*-values lower than 0.05 are highlighted in yellow.

Supplementary Data 6 | Gene ontology enrichment analysis of differentially expressed platelet proteins following exercise in young mice.

An Excel spreadsheet containing results of the gene ontology enrichment analyses for upregulated (Up) and downregulated platelet proteins (Down) in young running mice. BP: biological process; MF: molecular function.

Supplementary Data 7 | Gene ontology enrichment analysis of differentially expressed platelet proteins following exercise in aged mice.

An Excel spreadsheet containing results of the gene ontology enrichment analyses for upregulated (Up) and downregulated platelet proteins (Down) in aged mice following 4 days and 28 days of running. BP: biological process; MF: molecular function.

Supplementary Data 8 | Details of statistical analysis.

An Excel spreadsheet detailing the results of each statistical test performed in Graph Pad Prism.
